# Potential to Enhance the Prescribing of Generic Drugs in Patients with Mental Health Problems in Austria; Implications for the Future

**DOI:** 10.3389/fphar.2012.00198

**Published:** 2013-01-07

**Authors:** Brian Godman, Anna Bucsics, Thomas Burkhardt, Jutta Piessnegger, Manuela Schmitzer, Corrado Barbui, Emanuel Raschi, Marion Bennie, Lars L. Gustafsson

**Affiliations:** ^1^Division of Clinical Pharmacology, Karolinska University Hospital HuddingeStockholm, Sweden; ^2^Mario Negri Institute for Pharmacological ResearchMilan, Italy; ^3^Prescribing Research Group, University of Liverpool Management SchoolLiverpool, UK; ^4^Hauptverband der Österreichischen SozialversicherungsträgerWien, Austria; ^5^Section of Psychiatry, Department of Public Health and Community Medicine, WHO Collaborating Centre for Research and Training in Mental Health and Service Evaluation, University of VeronaVerona, Italy; ^6^Department of Medical and Surgical Sciences, Pharmacology Unit, Alma Mater Studiorum – University of BolognaBologna, Italy; ^7^Strathclyde Institute for Pharmacy and Biomedical Sciences, University of StrathclydeGlasgow, UK; ^8^Information Services Division, NHS National Services ScotlandEdinburgh, UK

**Keywords:** Austria, antidepressants, atypical antipsychotics, drug utilization studies, generics, risperidone, reforms, schizophrenia

## Abstract

**Background:** Scrutiny over pharmaceutical expenditure is increasing leading to multiple reforms. This includes Austria with measures to lower generic prices and enhance their utilization. However the situation for newer antidepressants and atypical antipsychotic medicines (AAPs) is different to PPIs, statins, and renin-angiotensin inhibitor drugs with greater tailoring of therapy and no wish to switch products in stable patients. Authorities welcome generics though given the high costs particularly of single-sourced AAPs. **Objective:** Assess (a) changes in utilization of venlafaxine versus other newer antidepressants before and after availability of generics, (b) utilization of generic versus originator venlafaxine, (c) price reductions of venlafaxine over time and their influence on total expenditure, (d) utilization of risperidone versus other AAPs, (e) suggest potential additional reforms that could be introduced if pertinent to further enhance the use of generics. **Methodology:** A quasi-experimental study design with a segmented time series and an observational study. Utilization measured in defined daily doses (DDDs) and total expenditure per DDD and over time. **Results:** No appreciable changes in the utilization of venlafaxine and risperidone after generics. The reduction in expenditure/DDD for venlafaxine decreased overall expenditure on newer antidepressants by 5% by the end of the study versus just before generics despite a 37% increase in utilization. Expenditure will further decrease if reduced prescribing of duloxetine. **Conclusion:** Depression, schizophrenia, and bipolar diseases are complex diseases. As a result, specific measures are needed to encourage the prescribing of generic risperidone and venlafaxine when multiple choices are appropriate. Authorities cannot rely on a “Hawthorne” effect between classes to enhance the use of generics. Measures may include prescribing restrictions for duloxetine. No specific measures planned for AAPs with more multiple-sourced AAPs becoming available.

## Background

### General

Scrutiny on pharmaceutical expenditure has intensified across Europe with its growth outstripping other components of ambulatory care, resulting in pharmaceutical expenditure becoming the largest or equaling the largest cost component in ambulatory care (Wettermark et al., 2008; Godman et al., 2008a, 2009a, 2010a, 2012a; Coma et al., 2009; Sermet et al., 2010; Voncina et al., 2011). This growth is set to continue unless addressed, driven by well known factors including changing demographics, rising patient expectations, strict clinical targets, and the continued launch of new premium priced medicines (Garattini et al., 2008; Wettermark et al., 2008; Godman et al., 2008a, 2009a, 2010a, 2012a; Coma et al., 2009; Sermet et al., 2010; Voncina et al., 2011).

These concerns have resulted in multiple reforms being instigated among countries to contain costs without compromising the quality of care (Godman et al., 2008a,b, 2009a,b, 2010b, 2011a,b, 2012b; Coma et al., 2009; Norman et al., 2009; Wettermark et al., 2009; McGinn et al., 2010; Sermet et al., 2010; Gustafsson et al., 2011; Voncina et al., 2011; Bennie et al., 2012). Austria is no exception. Multiple measures and initiatives introduced in Austria for existing medicines include pricing regulations for generics and originators once multiple-sourced products are available. Under these initiatives, the price of the third generic has to be at least 60% below single-sourced prices to be reimbursed (Godman et al., 2008a, 2009c, 2010a,c, 2011a). This price also establishes the reimbursed price for the originator (Godman et al., 2008a, 2009c, 2010a,c, 2011a). Thereafter, market forces act to obtain lower prices for successive branded generics. These include physician IT systems highlighting the cheapest multiple-sourced product, which build on quarterly information sent to physicians, physicians’ prescribing costs benchmarked against each other coupled with financial incentives to prescribe the cheapest multiple-sourced product, as well as quality circles among physicians with the objective to increase the prescribing of generics versus originators (Godman et al., 2008a, 2009c, 2010a,c, 2012a; Spiegel et al., 2012).

These multiple measures helped increase prescribing efficiency in drug classes [Anatomical Therapeutic Chemical Classification – ATC – Level 4; World Health Organization (WHO), 2003] where the products are seen as essentially similar in all or nearly all patients [Godman et al., 2008b, 2009a,c, 2010c, 2011a,b, 2012b; Voncina et al., 2011; McGinn et al., 2010; Gustafsson et al., 2011; Bennie et al., 2012]. Prescribing efficiency in this situation is defined as utilization growing at a faster rate than expenditure, with outcomes seen as similar between products. Pertinent drug classes include the proton pump inhibitors (PPIs), statins, and renin-angiotensin inhibitor drugs (Godman et al., 2009c, 2010c, 2011b, 2012b; Martikainen et al., 2010; McGinn et al., 2010; Gustafsson et al., 2011; Voncina et al., 2011; Bennie et al., 2012). The various measures, with their different intensities, resulted in the following outcomes in Austria (Godman et al., 2009c, 2010c, 2012b; Voncina et al., 2011):

• PPIs – utilization increased (Defined Daily Doses – DDDs) 3.6-fold between 2001 and 2007 (DDDs) but total expenditure only increased 2.1-fold
• Statins – utilization increased approximately 2.4-fold (DDDs) between 2001 and 2007 but there was a 3% decrease in total expenditure
• Angiotensin Converting Enzyme Inhibitors (ACEIs) and Angiotensin Receptor Blockers (ARBs) – utilization increased 69% between 2001 and 2007, with total expenditure increasing only 23%.

### Antidepressants

Newer antidepressants, including the dual acting antidepressants venlafaxine and mirtazapine, have also lost their patents during the past decade in Austria. Venlafaxine and mirtazapine are typically prescribed second line after the Selective Serotonin Re-uptake Inhibitors (SSRIs) with their increased effectiveness over the SSRIs (Yu-Isenberg et al., 2004; Baldomero et al., 2005; Agüera-Ortiz and Ramos Garcia, 2006; Cipriani et al., 2009). Among the newer antidepressants, duloxetine and reboxetine appear to be less effective than venlafaxine and mirtazapine (Cipriani et al., 2009).

Venlafaxine in Austria now includes generic immediate release (IR) and extended release (ER) formulations from May 2009. ER venlafaxine was launched to reduce possible side-effects associated with IR venlafaxine (Yu-Isenberg et al., 2004; Baldomero et al., 2005; Agüera-Ortiz and Ramos Garcia, 2006). Consequently, there should be an opportunity to save resources with the instigation of various measures to lower the prices of generics and the originator once multiple-sourced products become available as well as encourage their prescribing.

However unlike the PPIs, statins and renin-angiotensin inhibitor drugs, newer antidepressants should not be considered as a single class as there are differences between them both in terms of their effectiveness and side-effects (Cipriani et al., 2009). In view of this, it is acknowledged that patients should not be switched between therapies if they are responding to a particular antidepressant.

There is no reason though why patients should not be prescribed a multiple sourced antidepressant when suitable choices are available. This is especially the case when physicians are being encouraged through a variety of measures to preferentially prescribe generics (Godman et al., 2008a).

### Antipsychotics

The utilization of atypical antipsychotic drugs has also been increasing in recent years for the management of schizophrenia and bipolar disorders (Knapp et al., 2005; Mirandola et al., 2006; NICE, 2008; Crystal et al., 2009; Wladysiuk et al., 2011). As a result of their increased prescribing, worldwide sales of atypical antipsychotic drugs were over $US5bn per year in the early 2000s, reaching $14.6bn in the US alone in 2009 (Andretta et al., 2005; Wladysiuk et al., 2011; Leslie and Rosenheck, 2012).

The increased prescribing of atypical antipsychotic medicines (AAPs) appears to have been driven by meta-analyses and other studies suggesting greater efficacy and functional recovery, as well as lower side-effects than typical antipsychotics and other drugs (Mond et al., 2003; Knapp et al., 2005; Leucht et al., 2009; Department of Justice settlement agreement, 2010; Alexander et al., 2011; Kishimoto et al., 2011; Fisk et al., 2012). These clinical improvements resulted in reports of improved compliance and persistence for atypical antipsychotics, although others have not seen this in practice (Taylor et al., 2003; Valenstein et al., 2004; Ren et al., 2006). As a result, in June 2002, the National Institute for Health and Clinical Excellence (NICE) in the UK recommended the use of atypical (newer) oral antipsychotic drugs in people with newly diagnosed schizophrenia and those currently taking typical (older) antipsychotics where their symptoms are being controlled but have problems with side-effects (Walley, 2004). NICE recently updated its guidance for patients with newly diagnosed schizophrenia offered oral antipsychotic medication (NICE, 2009), stating that healthcare professionals should “*provide information and discuss the benefits and side-effect profile of each drug with the service user. The choice of drug should be made by the service user and healthcare professional together, considering*:

• *the relative potential of individual antipsychotic drugs to cause extrapyramidal side-effects (including akathisia), metabolic side-effects (including weight gain), and other side-effects (including unpleasant subjective experiences)*
• *the views of the carer where the service user agrees.”*

However, there have been concerns about the quality of the evidence and the extent of the differences in daily practice with the effectiveness of typical versus atypical antipsychotics (Walley, 2004). There has also been growing criticism that high-potency haloperidol (typical antipsychotic) was used as a comparator in the studies with second generation (atypical antipsychotics) thereby biasing the results as likely to be associated with a high rate of extrapyramidal side-effects, and only a limited number of studies used medium-potency first generation (typical) antipsychotics as the comparator (Leucht et al., 2003; Tyrer and Kendall, 2009). Alongside this, greater levels of side-effects such as weight gain, hyperlipidemia, and Type 2 diabetes with atypical antipsychotics (Andretta et al., 2005; Gardner et al., 2005; NICE, 2009; Tyrer and Kendall, 2009; Wladysiuk et al., 2011). In addition, the risk of QT prolongation and subsequent arrhythmia-related events, i.e., *Torsade de Pointes* (TdP) and Sudden Cardiac Death, is increasingly seen as an important safety aspect to consider when atypical antipsychotic drugs are being prescribed (Titier et al., 2005; Haddad and Sharma, 2007). In the past, atypical antipsychotics have been perceived as generally having a favorable cardiac safety profile compared with typical antipsychotic drugs. However, these beliefs have been undermined by a recent cohort study finding a dose-dependent increased risk of sudden cardiac deaths among current users of atypical antipsychotic drugs (Ray et al., 2009), further corroborated by different case series (Vieweg et al., 2009) as well as pharmacovigilance analyses (Poluzzi et al., 2009; Meyer-Massetti et al., 2011), showing similar reporting ratios between typical and atypical antipsychotic drugs in clinical practice. Tiihonen et al. (2009) have also raised concerns regarding the prescribing of quetiapine. In a recent study, the highest risk of mortality in patients with schizophrenia prescribed atypical antipsychotic drugs was with quetiapine, and the lowest was with clozapine. Haloperidol and risperidone had slightly lower adjusted hazard ratios than quetiapine (Tiihonen et al., 2009).

These debates have continued with the publication of the various findings from the Clinical Antipsychotic Trials of Intervention Effectiveness (CATIE) study in the US showing limited differences in effectiveness between the various antipsychotics, although this is not without criticism (Lieberman et al., 2005; Lieberman and Stroup, 2011; Wladysiuk et al., 2011; Berkowitz et al., 2012). The studies do show though that the variation in the effectiveness of the different AAPs can be substantial between individual patients, and that side-effects can also differ between different AAPs (Lieberman and Stroup, 2011). Consequently, “*treatments for schizophrenia must be individualized*” (Lieberman and Stroup, 2011). These findings are endorsed by Cochrane Collaboration reviews and other studies suggesting tentatively that olanzapine may be somewhat more efficacious than some other second generation antipsychotic drugs although concerns with high drop-out rates in the studies, with mixed results with risperidone (Hargreaves and Gibson, 2005; Heres et al., 2006; Komossa et al., 2010, 2011).

Other authors believed the modest gains with atypical antipsychotic drugs that have reported do not adequately reflect the improvements in quality of life perceived by patients, clinicians, or carers (Magnus et al., 2005), leading to increased use. Consequently, it is likely there will continue to be growing utilization of atypical antipsychotic medicines (Knapp et al., 2005; Mirandola et al., 2006; NICE, 2008; Crystal et al., 2009; Wladysiuk et al., 2011).

As a result, the availability of generic atypical antipsychotic medicines at low prices should be welcomed by health authorities and health insurance agencies with growing resource pressures. The first atypical antipsychotic drug to become available among Western countries was clozapine, with published studies showing no difference in outcomes between the originator and generics in practice once the bioavailability problem with the first generic clozapine in the US had been resolved (Bazire and Burton, 2004; Healy et al., 2005; Alessi-Severini et al., 2006; Paton, 2006; Araszkiewicz et al., 2008). More recently, generic olanzapine and generic risperidone became available among European countries. Again, there appears to be no patient issues in practice (Araszkiewicz et al., 2008; Khorana et al., 2011; Wladysiuk et al., 2011; Correl and Carbon, 2012), although some authors are more cautious (Desmarais et al., 2011; Correl and Carbon, 2012).

However, it is recognized that schizophrenia and other conditions such as bipolar disorders are complex diseases to treat. In addition, atypical antipsychotic drugs should not be considered as one class (Heres et al., 2006; NICE, 2009; Lieberman and Stroup, 2011). As a result, greater necessity for tailoring treatments depending on the patient and their characteristics, including current co-morbidities, having considered the safety profile of each drug including its pharmacokinetic profile (NICE, 2009; Poluzzi et al., 2009; Ray et al., 2009; Tiihonen et al., 2009; Vieweg et al., 2009; Lieberman and Stroup, 2011; Meyer-Massetti et al., 2011). In addition, switching patients between treatments should never be considered if they are stable on a particular atypical antipsychotic drug.

## Objectives

The availability of oral generic newer antidepressants as well as atypical antipsychotic drugs in addition to clozapine should in theory lead to their increased prescribing. However, this remains to be seen due to the complexities of treating depression, schizophrenia, and other mental health conditions compared with treating stomach related acid disorders, hypercholesterolemia or hypertension.

Consequently, the objectives of this paper are to assess (a) changes in the utilization of venlafaxine versus other newer antidepressants before and after the availability of generic IR and ER formulations, (b) utilization of generic versus originator venlafaxine, (c) price reductions of venlafaxine IR and ER over time and the subsequent influence on total expenditure of antidepressants over time, (d) the utilization of risperidone versus other atypical antipsychotic drugs over time following generic availability, (e) suggest potential additional reforms if pertinent that could be introduced in Austria to further enhance the prescribing of multiple-sourced antidepressants and atypical antipsychotic drugs when multiple drug choices are available and appropriate.

## Methodology

Two methodologies were utilized. The first involves a quasi-experimental study design with a segmented time series (Grimshaw et al., 2000; Wagner et al., 2002) to analyze retrospectively monthly reimbursed prescriptions for all patients in Austria covered by the social health insurance system prescribed at least one newer antidepressant including mirtazapine (N06AX11), venlafaxine (N06AX16), reboxetine (N06AX18); duloxetine (N06AX21) and agomelatine [N06AX22; World Health Organization (WHO), 2012] from May 2007, i.e., 24 months before the availability of generic venlafaxine in May 2009 (IR and ER) to August 2011, 27 months after generic venlafaxine. Desvenlafaxine (N06AX23) was never reimbursed in Austria. The data source was the internal data warehouse of the HVB (BIG), Cube HMSTAT, based on the “maschinelle Heilmittelabrechnung,” which covers approximately 98% of the Austrian population (Godman et al., 2009c, 2010c).

The second study is a retrospective observational study of the same population dispensed at least one atypical antipsychotic drug [N05AH03–05, N05AL05, N05AX08, 11–13; World Health Organization (WHO), 2003] between 2005, i.e., 1 year after generic risperidone became available, to 2010, 6 years after generic risperidone became available. Clozapine was not included in the analysis as it is reserved for resistant patients due to its side-effect profile (Healy et al., 2005; Paton, 2006; Leucht et al., 2009, 2011; Asenjo Lobos et al., 2010; Raja, 2011; Wladysiuk et al., 2011). The data source was again the internal data warehouse of the HVB (BIG), Cube HMSTAT, based on the “maschinelle Heilmittelabrechnung,” which covers approximately 98% of the Austrian population (Godman et al., 2009c, 2010c). No analysis of expenditure was undertaken as generic risperidone was already available by 2005 and reduced expenditure for risperidone is guaranteed given the current strict regulations governing the prices of generics and originators in Austria once multiple sourced products become available (Godman et al., 2008a, 2009c, 2010a,c, 2011a, 2012a).

The two methodologies were chosen to provide different data sets, especially since generic risperidone was already available before the study period.

Utilization was collated in terms of DDDs, with DDDs defined as “*the average maintenance dose of a drug when used in its major indication in adults,”* as this measure is recognized as the international standard to assess utilization patterns within and between countries [World Health Organization (WHO), 2012]. 2011 DDDs were used in line with international guidance [Vlahovic-Palcevski et al., 2010; Godman et al., 2010b, 2011a; World Health Organization (WHO), 2003, 2012].

The regression analysis for the newer antidepressants was undertaken using the “R” methodology (R Core Team, 2012). Using this methodology, reimbursed prescriptions were explained by a variable “Time” with its origin in May 2009 (generic venlafaxine) and a variable “Inter” to model the change in slope following the availability of generic venlafaxine.

Total costs in Euros were again used for the analysis to facilitate comparisons with previous studies (Godman et al., 2009c, 2010c). This is because it is difficult in practice to disaggregate pharmacy and wholesaler mark-ups from total costs in Austria, compounded by 20–25% of the Austrian population currently exempt from basic co-payment. As a result, total costs provide a more robust measure than estimating reimbursed costs using any derived formula (Godman et al., 2009c, 2010c). Total costs are the price paid to the pharmacy for the product including the ex-factory price, the wholesaler, and pharmacy mark-ups but excluding VAT (Godman et al., 2009c, 2010c). Total expenditure per DDD was computed for generic and originator IR and ER venlafaxine, as well as total monthly expenditure of the newer antidepressants over time.

There has been no allowance for inflation as we wanted to compute the actual influence of the various policies on total expenditure, as well as total expenditure/DDD, over time for the various antidepressants. In addition, the tendency of authorities across Europe is to cut prices of both patented (single-sourced) drugs and generics when pharmaceutical expenditure is rising more rapidly than target budgets (Coma et al., 2009; Sermet et al., 2010; Godman et al., 2010b, 2011a; Garuoliene et al., 2011; Vogler et al., 2011; Voncina et al., 2011). Alongside this, a number of European countries establish their initial prices for generics based on originator pre-patent loss prices, i.e. singles-sourced prices, which includes Austria (Godman et al., 2008a, 2009c, 2010a,c, 2011a,b, 2012a,c; Voncina et al., 2011). Consequently the use of total expenditure, as well as no allowance for inflation, is in line with previous publications.

## Results

The utilization of newer antidepressants increased by 37% from the launch of generic venlafaxine (IR and ER) until the end of the study based on accumulated six monthly DDDs (Table 1).

**Table 1:** **Utilization of newer antidepressants (mn DDDs) on an accumulated six monthly basis before and after the availability of generic venlafaxine ER (May 2009)**.

Months after generic venlafaxine	Six monthly accumulated data (DDDs mn)
18 months before	11,752
12 months before	12,701
6 months before	13,379
Launch generic venlafaxine	14,751
6 months after	15,804
12 months after	17,279
18 months after	18,120
24 months after	19,601
27 months after	20,238

**Figure 1:**
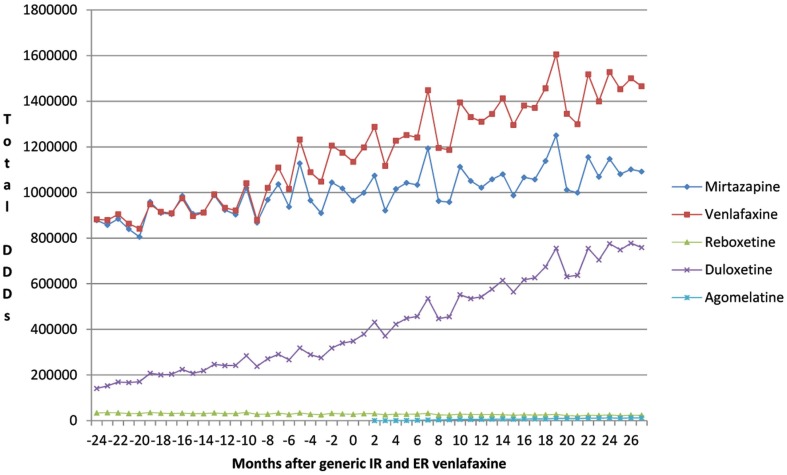
**Utilization pattern of newer antidepressants in DDDs before and after generic IR and ER venlafaxine (May 2009)**.

This growth included increasing utilization of venlafaxine (Figure 1), although there was no change in the overall utilization pattern of venlafaxine before and after the availability of generic IR and ER venlafaxine (Figure 1) with a probability of 0.591. This growth in the utilization of newer antidepressants (Table 1) is principally driven by increasing use of duloxetine in recent years, with the utilization of both reboxetine and agomelatine remaining low throughout the study period (Figure 1). As a result, the utilization of venlafaxine as a % of total antidepressant utilization decreased from 46% of all DDDs just before generic ER venlafaxine to 44% by August 2011.

The increase in the utilization of the other antidepressants combined was significant at the 0.1 level (*p* = 0.0843), but not at the 0.05 level (Figure 1, Table 2).

**Table 2:** **Residuals and coefficients for the change in the utilization of other antidepressants before and after generic ER venlafaxine**.

**RESIDUALS**				
Minimum	First Qtr	Median	Third Qtr	Maximum
−153307	−52437	−4846	54932	264627
Coefficients	Estimate	Standard error	*T* value	Significance
Intercept	1386886	26892	51.572	<0.001
Time	14276	2125	6.719	<0.001
Inter	6010	3411	1.762	0.0843

*Residual standard error: 91070 on 49 degrees of freedom. Multiple *R*-squared: 0.901, Adjusted *R*-squared: 0.897. *F*-statistic: 223.1 on 2 and 49 DF. *p*-Value < 2.2e − 16*.

**Figure 2:**
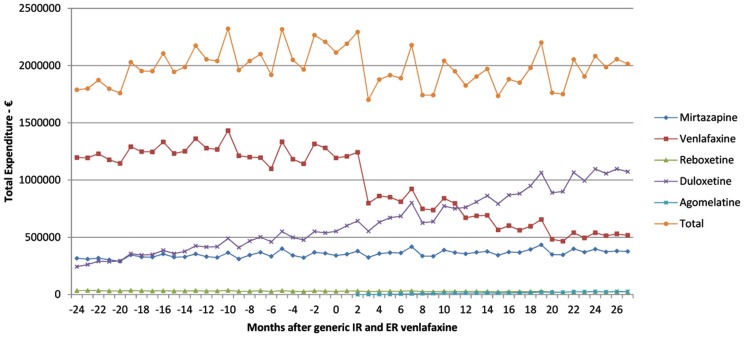
**Total expenditure on antidepressants in Austria before and after the availability of generic ER venlafaxine (May 2009)**.

There was a 5% reduction in total expenditure of the newer antidepressants 27 months after the availability of generic IR and ER venlafaxine at €12.1mn (accumulated 6 months) versus €12.725mn just before generic venlafaxine (accumulated 6 month basis). This was helped by a reduction in expenditure for venlafaxine after generic availability (Figure 2).

**Figure 3:**
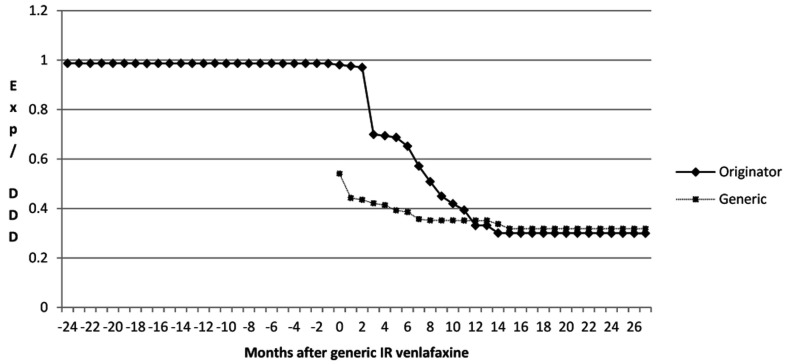
**Total expenditure/DDD for venlafaxine IR over time (€)**.

The reduction in expenditure for venlafaxine was helped by a reduction in expenditure/DDD for both IR and ER venlafaxine once generics became available (Figure 3). Total expenditure/DDD for generic IR venlafaxine in August 2011 was 68% below single-sourced prices (Figure 3), with a similar reduction in the price of the originator. There was a lower reduction in expenditure/DDD for generic ER venlafaxine ER (42% reduction) from May 2009 to August 2011.

**Figure 4:**
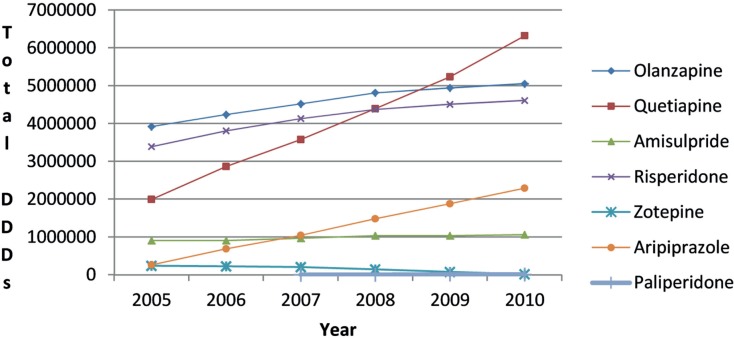
**Utilization of atypical antipsychotic drugs in Austria 2005–2010 (DDDs)**.

There was increased utilization of risperidone after the availability of generic risperidone (Figure 4). However, the 81% growth in overall atypical antipsychotic utilization from 2005 to 2010 (DDD basis) was principally driven by increasing utilization of quetiapine and aripiprazole (Figure 4). This resulted in the utilization of risperidone as a percentage of total atypical antipsychotic DDDs decreasing from 32% in 2005 to 24% in 2010.

## Discussion and Conclusion

We believe the lack of change in the utilization pattern of venlafaxine after the availability of generic IR and ER venlafaxine (Figure 1) reflects the greater complexity in treating patients with depression than those with stomach acid related disorders, hypercholesterolemia or hypertension with PPIs, statins, and renin-angiotensin inhibitor drugs respectively (Godman et al., 2009c, 2010c). Consequently, specific measures will be required to encourage the prescribing of multiple-sourced antidepressants when options are available to the prescribing physician. A similar situation is seen in the management of Parkinson’s disease where patients require add-on therapies to prolong the effectiveness and compliance with their treatment (Brkicic et al., 2012). As a result, there is again a need for specific demand-side measures to change subsequent prescribing habits with greater individualization of treatment (Brkicic et al., 2012). Consequently, authorities cannot rely on a Hawthorne effect between classes (Holden, 2001; Verstappen et al., 2004; Trietsch et al., 2009; Konstantinou, 2012) especially for complex disease areas. The Hawthorne effect in studies relates to the confounding that occurs if experimenters fail to realize how the consequences of a given subject’s performance in one area may affect activities in another area (Parsons, 1974).

Specific measures in the case of antidepressants could include academic detailing, prescribing guidance, and prescribing restrictions to encourage the prescribing of multiple-sourced antidepressants such as venlafaxine and mirtazapine where appropriate. Prescribing restrictions have been successfully introduced in Austria to limit the prescribing of atorvastatin and rosuvastatin versus generic statins, ARBs versus ACEIs, and single-sourced ARBs versus generic losartan (Godman et al., 2009c, 2010c, 2012b; Voncina et al., 2011; Bucsics et al., 2012). They have also successfully been instigated in other countries, although care is needed with follow-up to avoid disappointment (Godman et al., 2011b, 2012b).

Prescribing restrictions are an option for duloxetine (Figure 1) given its appreciably higher acquisition costs than multiple-sourced venlafaxine combined with published studies demonstrating lower effectiveness than other antidepressants (Cipriani et al., 2009). The restrictions would limit the prescribing of duloxetine to second line after mirtazapine or venlafaxine if there are concerns with their side-effects or effectiveness in practice. In addition intolerance to either mirtazapine or venlafaxine, which mirrors the situation for ARBs versus ACEIs in Austria (Godman et al., 2010c). We accept that the heterogeneous nature of the therapeutic indications for duloxetine, including diabetic peripheral neuropathic pain, may explain some of its increased utilization in recent years (Figure 1). However, it is difficult to know the extent of prescribing in these other indications without access to specific patient data. Prescribing restrictions have already been introduced for duloxetine in Sweden in similar circumstances [Cymbalta (duloxetine) receives restricted reimbursement, 2010]. As a result, providing an example for the authorities in Austria to follow if wished.

The reduction in expenditure/DDD for IR venlafaxine over time for both generics and the originator (Figure 3) is in line with expectations. This reduction mirrors the 83% reduction in expenditure/DDD for generic losartan in Austria by August 2011 versus single-source prices, generic ACEIs up to 77% below single-source prices, generic omeprazole 77% below, and generic simvastatin 72% below single-source prices by the end of 2007 in Austria (Godman et al., 2009c, 2010c). The reduction in expenditure/DDD for venlafaxine following the availability of multiple-sourced products helped the authorities reduce their expenditure on newer antidepressants in recent years. This was despite their utilization increasing by 37% from the availability of generic IR and ER venlafaxine to the end of the study (accumulated six monthly basis – Table 1). However, the reduction in total expenditure would have been greater without increasing expenditure on duloxetine in recent months (Figure 2).

There was no appreciable change in the utilization pattern for risperidone following the availability of generic risperidone (Figure 4). This is similar to the situation in Belgium, Scotland, Spain, and Ireland with again no appreciable change in the utilization of risperidone following the availability of generic risperidone (Godman et al., 2012d). This may reflect the advice from organizations such as NICE in the UK, as well as the conclusions from the various Cochrane reviews and the CATIE studies, that treatment with atypical antipsychotics should be tailored to the individual (Walley, 2004; Lieberman et al., 2005; Lieberman and Stroup, 2011; NICE, 2009; Komossa et al., 2010, 2011). Again this is unlike the situation with the PPIs, statins or renin-angiotensin inhibitor drugs, with schizophrenia and bipolar diseases seen as more complex to treat than acid-related stomach disorders, hypercholesterolemia or hypertension. Consequently, again no Hawthorne effect transferring initiatives from other disease areas to schizophrenia or bipolar disease (Holden, 2001; Verstappen et al., 2004; Trietsch et al., 2009; Konstantinou, 2012). The lack of a Hawthorne effect is no doubt enhanced in this situation by lack of any desire among physicians to switch patients between atypical antipsychotic drugs when they are stable on a particular one. As a result, specific measures will be needed to encourage the prescribing of multiple sourced antipsychotic drugs when several options are available to physicians to slow down the growth in the utilisation of single-source atypical antipsychotic drugs (Figure 4). Specific demand-side measures could include new guidelines highlighting the preferential prescribing of generic atypical antipsychotic drugs first line if there are no major patient issues or prescribing restrictions. However, no additional measures are currently being planned by the HVB with both quetiapine and olanzapine now available as multiple-sourced products in Austria. This may change though with increasing recognition that there can be limited clinical benefit of atypical antipsychotic drugs in some patients, alongside potentially life-threatening events (Titier et al., 2005; Maher et al., 2011), and with the possibility of new atypical antipsychotic drugs in the future. However, this remains to be seen.

Finally, there appears to be no problems in practice with either multiple-sourced venlafaxine (IR and ER) or oral risperidone. However, specific research will be needed before we can make any definitive statements.

In conclusion, depression, schizophrenia and bipolar diseases are complex diseases to treat with physicians having no wish to switch patients between medicines, especially when patients are stable on a particular medicine. As a result, specific measures are needed to encourage the prescribing of multiple-sourced antidepressants or atypical antipsychotic drugs when several options are available and appropriate, and authorities cannot rely on a “Hawthorne” effect between classes. Specific measures may include guidelines or prescribing restrictions for duloxetine, especially given concerns with its effectiveness versus other newer antidepressants such as venlafaxine and appreciably higher acquisition costs. No specific measures are currently being planned for generic atypical antipsychotic drugs in Austria with more multiple-sourced choices becoming available. However, the situation will be monitored.

## Conflict of Interest Statement

Anna Bucsics, Thomas Burkhardt, Jutta Piessnegger, and Manuela Schmitzer are employed by the HVB in Austria. The authors have no other conflicts of interest to declare. Thomas Burkhardt has now moved to Wiener Gebietskrankenkasse (WGKK) in Austria.
